# Users of rehabilitation services in 14 countries and territories affected by conflict, 1988–2018

**DOI:** 10.2471/BLT.19.249060

**Published:** 2020-07-08

**Authors:** Cornelia A Barth, Andreas Wladis, Catherine Blake, Prashant Bhandarkar, Cliona O’Sullivan

**Affiliations:** a International Committee of the Red Cross, 19 avenue de la paix, 1202 Geneva, Switzerland.; b Department of Biomedical and Clinical Sciences, Linköping University, Linköping, Sweden.; c School of Public Health, Physiotherapy and Sports Science, University College Dublin, Dublin, Ireland.; d Doctors For You, Mumbai, India.

## Abstract

**Objective:**

To analyse the demographic and clinical characteristics of people attending physical rehabilitation centres run or supported by the International Committee of the Red Cross in countries and territories affected by conflict.

**Methods:**

Of 150 such rehabilitation centres worldwide, 38 use an electronic patient management system. We invited all 38 centres to participate. We extracted de-identified data from 1988 to 2018 and categorized them by sex, age, country or territory and reason for using rehabilitation services.

**Findings:**

Thirty-one of the 38 rehabilitation centres in 14 countries and territories participated. We included data for 287 274 individuals. Of people using rehabilitation services, 61.6% (176 949/287 274) were in Afghanistan, followed by 15.7% (44 959/287 274) in Cambodia. Seven places had over 9000 service users each (Afghanistan, Cambodia, Gaza Strip, Iraq, Myanmar, Somalia and Sudan). Overall, 72.6% (208 515/287 274) of service users were male. In eight countries, more than half of the users were of working age (18–59 years). Amputation was the most common reason for using rehabilitation services; 33.3% (95 574/287 274) of users were people with amputations, followed by 13.7% (39 446/287 274) with cerebral palsy. The male predominance was greater in the population aged 18–34 years (83.1%; 71 441/85 997) and in people with amputations (88.6%; 84 717/95 574) but was evident across all places, age groups and health conditions.

**Conclusion:**

The considerably lower attendance of females at the rehabilitation centres highlights the need to understand the factors that affect the accessibility and acceptability of rehabilitation for women and girls in conflict settings.

## Résumé

**Objectif:**

Analyser les caractéristiques démographiques et cliniques des personnes qui fréquentent des centres de réadaptation physique gérés ou soutenus par le Comité international de la Croix-Rouge dans des pays et territoires touchés par des conflits.

**Méthodes:**

Trente-huit des 150 centres de réadaptation dans le monde utilisent un système de gestion électronique des patients. Nous les avons invités à participer. Nous avons prélevé des données dépersonnalisées issues d'une période comprise entre 1988 et 2018, puis nous les avons classées en fonction du sexe, de l'âge, du pays ou territoire, et du motif de recours aux services de réadaptation.

**Résultats:**

Sur les 38 centres de réadaptation, 31 centres répartis dans 14 pays ont participé. Nous avons inclus les données provenant de 287 274 individus. Parmi les personnes qui font appel à des services de réadaptation, 61,6% (176 949/287 274) résidaient en Afghanistan, et 15,7% (44 959/287 274) au Cambodge. Sept centres dénombraient plus de 9000 usagers chacun (Afghanistan, Bande de Gaza, Cambodge, Irak, Myanmar, Somalie et Soudan). Au total, 72,6% (208 515/287 274) des usagers de tels services étaient des hommes. Dans huit pays, plus de la moitié des usagers étaient en âge de travailler (18–59 ans). L'amputation était le motif le plus courant pour le recours aux services de réadaptation; 33,3% (95 574/287 274) des usagers avaient été amputés, suivis de 13,7% (39 446/287 274) souffrant de paralysie cérébrale. La prédominance masculine était surtout importante dans la tranche d'âge des 18–34 ans (83,1%; 71 441/85 997) et chez les personnes amputées (88,6%; 84 717/95 574), mais la tendance était manifeste quel que soit le lieu, l'âge et l'état de santé.

**Conclusion:**

La fréquentation féminine nettement plus faible observée dans les centres de réadaptation met en relief la nécessité d'identifier les facteurs qui affectent l'accessibilité et l'acceptation d'une réadaptation pour les femmes et filles en situation de conflit.

## Resumen

**Objetivo:**

Analizar las características demográficas y clínicas de las personas que asisten a los centros de rehabilitación física que el Comité Internacional de la Cruz Roja dirige o apoya en los países y territorios afectados por los conflictos.

**Métodos:**

De los 150 centros de rehabilitación similares que existen en todo el mundo, 38 aplican un sistema electrónico de gestión de pacientes. Se invitó a los 38 centros a participar. Se obtuvieron datos no identificados de 1988 a 2018 y se clasificaron por sexo, edad, país o territorio y motivo del uso de los servicios de rehabilitación.

**Resultados:**

Participaron 31 de los 38 centros de rehabilitación de 14 países y territorios. Se incluyeron datos de 287 274 personas. De las personas que usaron los servicios de rehabilitación, el 61,6 % (176 949/287 274) estaban en Afganistán, seguido por el 15,7 % (44 959/287 274) en Camboya. En siete lugares había más de 9000 usuarios de servicios cada uno (Afganistán, Camboya, Iraq, la Franja de Gaza, Myanmar, Somalia y Sudán). En total, el 72,6 % (208 515/287 274) de los usuarios del servicio eran hombres. En ocho países, más de la mitad de los usuarios estaban en edad de trabajar (18 a 59 años). La amputación fue la razón más común para utilizar los servicios de rehabilitación; el 33,3 % (95 574/287 274) de los usuarios eran personas con amputaciones, seguidas por el 13,7 % (39 446/287 274) con parálisis cerebral. El predominio de los varones fue mayor en la población de 18 a 34 años (83,1 %; 71 441/85 997) y en las personas con amputaciones (88,6 %; 84 717/95 574), pero fue evidente en todos los lugares, grupos de edad y condiciones de salud.

**Conclusión:**

La asistencia notablemente inferior de las mujeres a los centros de rehabilitación destaca la necesidad de comprender los factores que afectan el acceso y la aceptación de la rehabilitación para las mujeres y las niñas en los entornos de conflicto.

## ملخص

الغرض تحليل الخصائص الديموغرافية والسريرية للأشخاص الذين يزورون مراكز إعادة التأهيل البدني، والتي تديرها أو تدعمها لجنة الصليب الأحمر الدولية في الدول والمناطق الواقعة تحت وطأة الصراع.

الطريقة من بين 150 مركزاً لإعادة التأهيل حول العالم، يستخدم 38 منها نظاماً إلكترونياً لإدارة المرضى. قمنا بدعوة جميع المراكز الـ 38 للمشاركة. وقمنا باستخراج بيانات غير محددة الهوية من 1988 إلى 2018، وتصنيفها حسب الجنس أو العمر أو الدولة أو الإقليم، إلى جانب سبب اللجوء إلى خدمات إعادة التأهيل.

النتائج شارك 31 مركزاً من مراكز إعادة التأهيل الـ 38 في 14 دولة وإقليم التي شاركت. وقمنا بتضمين بيانات 287274 شخصاً. من بين الأشخاص الذين يلجئون لخدمات إعادة التأهيل، كان 61.6٪ (176949/287274) منهم في أفغانستان، يليهم 15.7٪ (44959/287274) منهم في كمبوديا. وكان هناك سبع جهات يوجد بكل منها أكثر من 9000 مستخدم للخدمة (أفغانستان، وكمبوديا، وقطاع غزة، والعراق، وميانمار، والصومال، والسودان). وبوجه عام، كان 72.6٪ (208515/287274) من مستخدمي الخدمة من الذكور. وفي ثماني دول، كان أكثر من نصف المستخدمين في سن العمل (18 إلى 59 عاماً). كان البتر هو السبب الأكثر شيوعاً للاستعانة بخدمات إعادة التأهيل؛ حيث كان 33.3٪ (95574/287274) من المستخدمين من الأشخاص من ذوي حالات البتر، ويليهم 13.7٪ (39446/287274) من المصابين بالشلل الدماغي. كانت هيمنة الذكور أكبر في السكان الذين تتراوح أعمارهم من 18 إلى 34 عاماً (83.1٪؛ 71441/85997) وفي الأشخاص ذوي حالات البتر (88.6٪؛ 84717/95574)، ولكنها كانت واضحة في جميع الجهات والفئات العمرية والظروف الصحية .

الاستنتاج إن التردد الأقل، بشكل ملحوظ، للإناث على مراكز إعادة التأهيل يسلط الضوء على الحاجة إلى فهم العوامل التي تؤثر على إمكانية لجوء النساء والفتيات في ظروف الصراع لإعادة التأهيل، ومدى مقبولية ذلك.

## 摘要

**目的:**

旨在分析在受冲突影响的国家和地区人民中，接受红十字国际委员会运营或资助的身体康复中心服务的人群的人口和临床特征。

**方法:**

在全球 150 家此类康复中心中，有 38 家使用电子版的病人管理系统。我们邀请了这 38 家中心参与此次研究。我们提取了 1988 年到 2018 年间去除身份识别信息的数据，并将其按性别、年龄、国家或地区以及使用康复服务的原因进行分类

**结果:**

在这 14 个国家和地区的 38 家康复中心中，共有 31 家 参与此次研究。我们共采集了 287,274 份个人数据。在接受康复服务的人群中，阿富汗人占 61.6% (176,949/287,274)，柬埔寨人占 15.7% (44,959/287,274)。服务用户超过 9000 人的有 7 处（阿富汗、加沙地带、柬埔寨、缅甸、苏丹、索马里和伊拉克）。整体而言，72.6% (208,515/287,274) 的服务用户为男性。在 8 个国家中，超过一半的用户处于工作年龄（18-59 岁）。截肢是接受康复服务最常见的原因，33.3% (95,574/287,274) 的用户是截肢者，其次是脑瘫患者，比例为 13.7% (39,446/287,274)。男性明显居多的现象体现在 18-34 岁人群 (83.1%; 71,441/85,997) 和截肢者 (88.6%; 84 717/95 574) 中，但在所有地方、年龄组和健康状况中男性均占多数。

**结论:**

女性到康复中心参加治疗的比例相当低，这突出强调了需要了解处于冲突环境中的妇女和女童获得和接受康复的影响因素

## Резюме

**Цель:**

Анализ демографических и клинических показателей лиц, которые посещают центры физической реабилитации, находящиеся в ведении Международного комитета Красного Креста или поддерживаются им в странах и на территориях, затронутых конфликтом.

**Методы:**

Из 150 подобных реабилитационных центров по всему миру 38 используют электронную систему контроля пациентов. Мы пригласили все 38 центров принять участие в исследовании. Мы извлекли обезличенные данные за период с 1988 по 2018 гг. и классифицировали пациентов по полу, возрасту, стране или территории, а также по причине использования услуг по реабилитации.

**Результаты:**

Участие принял тридцать один из 38 реабилитационных центров 14 стран и территорий. Мы включили данные 287 274 человек. Из лиц, пользующихся услугами по реабилитации, 61,6% (176 949/287 274) проживали в Афганистане, на втором месте с показателем 15,7% (44 959/287 274) были пациенты из Камбоджи. В семи локациях насчитывалось более 9000 пользователей услуг по реабилитации (Афганистан, Ирак, Камбоджа, Мьянма, Сектор Газа, Сомали и Судан). Из числа включенных в исследование лиц 72,6% (208 515/287 274) пользователей услуг оказались мужчинами. В восьми странах более половины пользователей были трудоспособного возраста (18–59 лет). Ампутация была наиболее распространенной причиной использования услуг по реабилитации; 33,3% (95 574/287 274) пользователей были лица с ампутированными конечностями, второе место с показателем 13,7% (39 446/287 274) занимала группа пациентов с церебральным параличом. Более значительное преобладание мужчин наблюдалось среди населения в возрасте 18–34 лет (83,1%; 71 441/85 997), а также среди лиц с ампутированными конечностями (88,6%; 84 717/95 574). Указанное наблюдение было верно для всех локаций, возрастных групп и состояния здоровья.

**Вывод:**

Значительно более низкая посещаемость реабилитационных центров женщинами демонстрирует необходимость понимания факторов, влияющих на доступность и приемлемость реабилитации для женщин и девушек в условиях военных конфликтов.

## Introduction

The effects of conflict on population health include increased injury rates coupled with a collapse of health systems.^1^ The consequences of weak health systems are more far-reaching and complex than the effects of conflict-caused injury and physical impairment.^2^ The lack of disease prevention and health promotion services and good-quality health care increase the number of people with disabilities requiring rehabilitation.

While early rehabilitation has received more attention recently,^3^^–^^5^ the needs of people with permanent disabilities for continuing and costly rehabilitation and assistive technology in conflict settings have not been adequately addressed.^6^^,^^7^

The Physical Rehabilitation Programme of the International Committee of the Red Cross (ICRC) aims to bridge the gap between immediate humanitarian rehabilitation needs and long-term programming for people with disabilities in difficult environments.^8^ Over the programme’s 40-year history, the number of projects has increased across many countries in response to population rehabilitation needs during and after conflict.

Rehabilitation in all physical rehabilitation centres supported by the ICRC consists of assistive technology and physiotherapy. Over the past 30 years and notably after adoption of the United Nations *Convention on the Rights of Persons with Disabilities* in 2006,^9^ most physical rehabilitation centres broadened their scope and employed, or referred people to, professionals offering psychosocial support, disability sports and educational or professional (re)integration. In addition, increasing numbers of trained prosthetists, orthotists and physiotherapists have been working in clinics and supervising teams, which has contributed to the implementation of professional standards adapted to the context.^8^ A patient management system was introduced in 2001,^10^ a field-based software that allows physical rehabilitation centres to manage their user data.^11^

Research on people using rehabilitation services in fragile countries is limited.^12^^,^^13^ People with disabilities attending ICRC physical rehabilitation centres are particularly vulnerable because their disability is often combined with factors such as gender bias and poverty.^8^ Access to and availability of rehabilitation services during conflict is difficult because of reduced workforce, scarce resources and broken health systems.

Understanding the characteristics of people with disabilities who access physical rehabilitation centres is important to identify the main health conditions for which rehabilitation is sought and the affected populations with specific needs, so as to inform the development of rehabilitation systems in conflict settings. We aimed to examine existing data from patient management systems to determine the demographic characteristics and clinical presentations of people attending ICRC physical rehabilitation centres in different countries.

## Methods

### Study design

This was a retrospective descriptive study of aggregated data from patient management systems for all people seeking rehabilitation services from 1988 to 2018 who were registered in participating physical rehabilitation centres. Data on people seeking services before the introduction of the patient management system had been manually transferred onto the system from the paper records. The timespan varied between physical rehabilitation centres depending on when the ICRC support began and when the patient management system was introduced (Table 1).

**Table 1:** Characteristics of rehabilitation centres included in the study on people using rehabilitation services in 14 countries and territories affected by conflict, 1988–2018

Country or territory	No. of physical rehabilitation centres	ICRC support since	Use of patient management system	History of conflict	User records, no. (%)
Afghanistan	7^a^	1987	1988–2018	Ongoing	176 949 (61.6)
Cambodia	2^b^	1991	1991–2018	Post-conflict	44 959 (15.7)
Iraq	1^a^	1994	1996–2018	Ongoing; hosting refugees	13 749 (4.8)
Myanmar	5^b^	1985^c^	2002–2018	Ongoing	10 498 (3.7)
Sudan	2^b^	1985^c^	2000–2018	Ongoing; hosting refugees	9 683 (3.4)
Somalia	3^b^	2016	2016–2018	Ongoing	9 081 (3.2)
Gaza Strip	1^b^	1989^c^	1991–2018	Ongoing	9 029 (3.1)
Pakistan	1^b^	1981^c^	2005–2018	Ongoing; hosting refugees	4 608 (1.6)
Ethiopia	2^b^	1979^c^	2007–2018	Hosting refugees; post-conflict	3 445 (1.2)
Democratic Republic of the Congo	2^b^	1998	2007–2018	Ongoing	2 587 (0.9)
Togo	1^b^	2010	2015–2018	Hosting refugees	1 142 (0.4)
Niger	2^b^	2010	2012–2018	Hosting refugees	922 (0.3)
Syrian Arab Republic	1^a^	1983^c^	2015–2018	Ongoing	415 (0.1)
Algeria	1^b^	2002	2008–2018	Hosting refugees	207 (0.1)
Total	31	NA	1988–2018	NA	287 274 (100.0)

ICRC: International Committee of the Red Cross; NA: not applicable.

^a^ ICRC-run centre.

^b^ ICRC-supported partner centre.

^c^ With short interruptions because of conflict, political situation and security.

Note: Ongoing means country with an ongoing protracted crisis with changing patterns of fighting and changing lines of demarcation throughout the years of data collection; hosting refugees means country that has hosted (and treated disabled) refugees from neighbouring conflict(s) throughout the years of data collection; post-conflict means country where the conflict has ended, which has been treating people with conflict-related disabilities throughout the years of data collection.

### Study setting

Physical rehabilitation centres partnered with the ICRC are advised, but not obliged to install the patient management system. At the time of data collection, 38 of 150 physical rehabilitation centres in 35 low-income and conflict-affected countries and territories had installed this system. The analysed data collection represents countries and territories, and physical rehabilitation centres that we consider were in the midrange of human and technological resource capacity to undertake electronic data collection. Outside this range were: (i) centres that had difficulty using software (e.g. centres with badly affected electricity and internet infrastructure, or which lacked, or had a high turnover of, staff with information technology skills), and (ii) centres in countries and territories with more advanced health information systems than the patient management system. Some centres were not using the patient management system because of data protection policies and the perceived sensitivity of user files, despite guaranteed anonymity of the data.

We wrote to the managers of all 38 physical rehabilitation centres using the patient management system explaining the purpose of the study in detail and inviting them to participate and provide us with de-identified data.

### Study population

The study population was newly registered users of rehabilitation services at the physical rehabilitation centres. On registration, demographic and clinical characteristics of the person seeking rehabilitation services are recorded as part of routine documentation.

### Data collection

We retrieved data on the country or territory where the centre was located, and age and sex of the users and their main reason for attending the centre. The main reasons for attending the centre included a mix of symptoms (e.g. muscle weakness), causes (e.g. ageing), diseases (e.g. encephalitis), injuries (e.g. burns) or disorders (e.g. cerebral palsy).

Data accuracy depended on the quality of self-reported information provided by the person attending the rehabilitation centre and the recorded observations of the staff of the centre who have varying levels of professional training. We considered variables such as sex and age as robust. However, given the lack of medical personnel and diagnostic tests, recording an accurate clinical diagnosis can be difficult.

### Data analysis

We cleaned, merged and aggregated data by sex and age, and organized into the variables of interest. We categorized people according to age as: young child (younger than 5 years); child (5–17 years); young adult (18–34 years); adult (35–59 years); and older adult (older than 59 years). We grouped the main reasons for attending the centre into: musculoskeletal (amputation including congenital limb deficiency and fracture); neurological (paraplegia, tetraplegia or hemiplegia and sequelae of polio); and paediatric (congenital conditions: clubfoot and cerebral palsy) according to clinical group and age at registration.

We used descriptive statistics for our primary analysis using Microsoft Excel, 2016 (Microsoft, Redmond, United States of America). We summarized data as counts and percentages.

### Ethical approval

We received ethical exemption to conduct an analysis on de-identified data by the Commission Cantonale d’Ethique de la Recherche, Geneva, Switzerland (reference number: REQ-2019–00027). Data sharing agreements were approved by ICRC, Linköping University, Sweden and University College Dublin, Ireland.

## Results

### Rehabilitation centres

Of the 38 physical rehabilitation centres in low-income and conflict-affected countries and territories that had installed the patient management system, 31 from 14 countries and territories (Afghanistan, Algeria, Cambodia, Democratic Republic of the Congo, Ethiopia, Gaza Strip, Iraq, Myanmar, Niger, Pakistan, Somalia, Sudan, Syrian Arab Republic and Togo) participated in the study and provided data on 289 248 users. Seven centres could not participate because of challenges in data extraction during the study’s timeline, including remote physical rehabilitation centres without a permanent ICRC presence.

Minor problems in the patient management system software resulted in invalid entries for 1974 users, which we excluded. The problems were: missing diagnosis (6 records), missing date of birth or age recorded as < 0 and > 99 (1793 records) and double entries (175 records). Thus, 287 274 unique user sets of data were included in the analysis.

Table 1 gives information on the rehabilitation centres in the countries: number of centres, year the ICRC started to support the centres, duration of using the patient management system, history of conflict in the country and number of user records. Of the 14 countries and territories, seven had more than 9000 users (Afghanistan, Cambodia, Iraq, Myanmar, Somalia, Gaza Strip and Sudan, in descending order of the number of user records) comprising 95.4% (273 948/287 274) of the total data set. Afghanistan had 61.6% (176 949/287 274) of user records and Cambodia had 15.7% (44 959/287 274).

### Demographics

Table 2 shows the total user numbers by sex and age group. Table 3 shows the same data by country or territory. The tables show that overall, and in most places, considerably more service users were males (overall 72.6%; 208 515/287 274). A greater proportion of males used rehabilitation services than females also in Togo (51.5% male; 588/1142), Gaza Strip (56.6% male; 5112/9029) and Democratic Republic of the Congo (57.9% male; 1498/2587), but the differences were not large.

**Table 2:** Sex and age distribution of service users in 14 countries and territories affected by conflict, 1988–2018

Age group	Males			Females			Total
	No. (%)	% in age group		No. (%)	% in age group		No. (%)
Young child (< 5 years)	31 054 (14.9)	59.2		21 417 (27.2)	40.8		52 471 (18.3)
Child (5–17 years)	39 838 (19.1)	64.0		22 389 (28.4)	36.0		62 227 (21.7)
Young adult (18–34 years)	71 441 (34.3)	83.1		14 556 (18.5)	16.9		85 997 (29.9)
Adult (35–59 years)	48 902 (23.5)	78.9		13 075 (16.6)	21.1		61 977 (21.6)
Older adult (≥ 60 years)	17 280 (8.3)	70.2		7 322 (9.3)	29.8		24 602 (8.6)
**Total**	**208 515 (100.0)**	**72.6**		**78 759 (100.0)**	**27.4**		**287 274 (100.0)**

Note: We used data from International Committee of the Red Cross (ICRC)-owned and official ICRC-partner centres only.

**Table 3:** Sex and age distribution of service users in 14 countries and territories affected by conflict, 1988–2018

Country or territory, age group	Males			Females			Total
	No. (%)	% in age group		No. (%)	% in age group		No. (%)
**Afghanistan**	129 684 (100.0)	73.3		47 265 (100.0)	26.7		176 949 (100.0)
Young child	23 466 (18.1)	60.3		15 458 (32.7)	39.7		38 924 (22.0)
Child	29 784 (23.0)	65.9		15 416 (32.6)	34.1		45 200 (25.5)
Young adult	46 709 (36.0)	85.5		7 953 (16.8)	14.5		54 662 (30.9)
Adult	21 286 (16.4)	77.6		6 149 (13.0)	22.4		27 435 (15.5)
Older adult	8 439 (6.5)	78.7		2 289 (4.8)	21.3		10 728 (6.1)
**Algeria**	131 (100.0)	63.3		76 (100.0)	36.7		207 (100.0)
Young child	9 (6.9)	50.0		9 (11.8)	50.0		18 (8.7)
Child	16 (12.2)	66.7		8 (10.5)	33.3		24 (11.6)
Young adult	20 (15.3)	62.5		12 (15.8)	37.5		32 (15.5)
Adult	41 (31.3)	67.2		20 (26.3)	32.8		61 (29.5)
Older adult	45 (34.4)	62.5		27 (35.5)	37.5		72 (34.8)
**Cambodia**	32 609 (100.0)	72.5		12 350 (100.0)	27.5		44 959 (100.0)
Young child	1 497 (4.6)	56.3		1 163 (9.4)	43.7		2 660 (5.9)
Child	3 919 (12.0)	59.0		2 727 (22.1)	41.0		6 646 (14.8)
Young adult	11 682 (35.8)	81.2		2 712 (22.0)	18.8		14 394 (32.0)
Adult	12 295 (37.7)	80.7		2 944 (23.8)	19.3		15 239 (33.9)
Older adult	3 216 (9.9)	53.4		2 804 (22.7)	46.6		6 020 (13.4)
**Democratic Republic of the Congo**	1 498 (100.0)	57.9		1 089 (100.0)	42.1		2 587 (100.0)
Young child	414 (27.6)	55.2		336 (30.9)	44.8		750 (29.0)
Child	230 (15.4)	58.4		164 (15.1)	41.6		394 (15.2)
Young adult	383 (25.6)	62.5		230 (21.1)	37.5		613 (23.7)
Adult	366 (24.4)	60.8		236 (21.7)	39.2		602 (23.3)
Older adult	105 (7.0)	46.1		123 (11.3)	53.9		228 (8.8)
**Ethiopia**	2 534 (100.0)	73.6		911 (100.0)	26.4		3 445 (100.0)
Young child	101 (4.0)	68.2		47 (5.2)	31.8		148 (4.3)
Child	348 (13.7)	62.6		208 (22.8)	37.4		556 (16.1)
Young adult	995 (39.3)	68.0		469 (51.5)	32.0		1 464 (42.5)
Adult	776 (30.6)	83.7		151 (16.6)	16.3		927 (26.9)
Older adult	314 (12.4)	89.7		36 (4.0)	10.3		350 (10.2)
**Gaza Strip**	5 112 (100.0)	56.6		3 917 (100.0)	43.4		9 029 (100.0)
Young child	2 254 (44.1)	49.6		2 292 (58.5)	50.4		4 546 (50.3)
Child	1 127 (22.0)	52.2		1 031 (26.3)	47.8		2 158 (23.9)
Young adult	913 (17.9)	82.3		197 (5.0)	17.7		1 110 (12.3)
Adult	579 (11.3)	67.7		276 (7.0)	32.3		855 (9.5)
Older adult	239 (4.7)	66.4		121 (3.1)	33.6		360 (4.0)
**Iraq**	10 682 (100.0)	77.7		3 067 (100.0)	22.3		13 749 (100.0)
Young child	671 (6.3)	61.1		428 (14.0)	38.9		1 099 (8.0)
Child	1427 (13.4)	64.9		771 (25.1)	35.1		2 198 (16.0)
Young adult	3 450 (32.3)	83.3		690 (22.5)	16.7		4 140 (30.1)
Adult	3 843 (36.0)	84.5		703 (22.9)	15.5		4 546 (33.1)
Older adult	1 291 (12.1)	73.1		475 (15.5)	26.9		1 766 (12.8)
**Myanmar**	8 821 (100.0)	84.0		1 677 (100.0)	16.0		10 498 (100.0)
Young child	119 (1.3)	50.4		117 (7.0)	49.6		236 (2.2)
Child	480 (5.4)	58.9		335 (20.0)	41.1		815 (7.8)
Young adult	2 960 (33.6)	87.5		421 (25.1)	12.5		3 381 (32.2)
Adult	4 424 (50.2)	88.7		566 (33.8)	11.3		4 990 (47.5)
Older adult	838 (9.5)	77.9		238 (14.2)	22.1		1 076 (10.2)
**Niger**	594 (100.0)	64.4		328 (100.0)	35.6		922 (100.0)
Young child	77 (13.0)	56.2		60 (18.3)	43.8		137 (14.9)
Child	114 (19.2)	58.2		82 (25.0)	41.8		196 (21.3)
Young adult	170 (28.6)	68.5		78 (23.8)	31.5		248 (26.9)
Adult	181 (30.5)	69.9		78 (23.8)	30.1		259 (28.1)
Older adult	52 (8.8)	63.4		30 (9.1)	36.6		82 (8.9)
**Pakistan**	3 466 (100.0)	75.2		1 142 (100.0)	24.8		4 608 (100.0)
Young child	637 (18.4)	64.3		353 (30.9)	35.7		990 (21.5)
Child	411 (11.9)	65.0		221 (19.4)	35.0		632 (13.7)
Young adult	1 067 (30.8)	82.3		229 (20.1)	17.7		1 296 (28.1)
Adult	1 026 (29.6)	81.0		241 (21.1)	19.0		1 267 (27.5)
Older adult	325 (9.4)	76.8		98 (8.6)	23.2		423 (9.2)
**Somalia**	5 620 (100.0)	61.9		3 461 (100.0)	38.1		9081 (100.0)
Young child	1 139 (20.3)	61.8		704 (20.3)	38.2		1 843 (20.3)
Child	1 107 (19.7)	59.0		769 (22.2)	41.0		1 876 (20.7)
Young adult	1 150 (20.5)	64.1		645 (18.6)	35.9		1 795 (19.8)
Adult	1 242 (22.1)	62.9		733 (21.2)	37.1		1 975 (21.7)
Older adult	982 (17.5)	61.7		610 (17.6)	38.3		1 592 (17.5)
**Sudan**	6 866 (100.0)	70.9		2 817 (100.0)	29.1		9 683 (100.0)
Young child	417 (6.1)	61.1		265 (9.4)	38.9		682 (7.0)
Child	732 (10.7)	57.4		544 (19.3)	42.6		1 276 (13.2)
Young adult	1 771 (25.8)	68.0		833 (29.6)	32.0		2 604 (26.9)
Adult	2 600 (37.9)	76.6		794 (28.2)	23.4		3 394 (35.1)
Older adult	1 346 (19.6)	77.9		381 (13.5)	22.1		1 727 (17.8)
**Syrian Arab Republic**	310 (100.0)	74.7		105 (100.0)	25.3		415 (100.0)
Young child	9 (2.9)	60.0		6 (5.7)	40.0		15 (3.6)
Child	67 (21.6)	69.8		29 (27.6)	30.2		96 (23.1)
Young adult	108 (34.8)	78.8		29 (27.6)	21.2		137 (33.0)
Adult	96 (31.0)	75.6		31 (29.5)	24.4		127 (30.6)
Older adult	30 (9.7)	75.0		10 (9.5)	25.0		40 (9.6)
**Togo**	588 (100.0)	51.5		554 (100.0)	48.5		1 142 (100.0)
Young child	244 (41.5)	57.7		179 (32.3)	42.3		423 (37.0)
Child	76 (12.9)	47.5		84 (15.2)	52.5		160 (14.0)
Young adult	63 (10.7)	52.1		58 (10.5)	47.9		121 (10.6)
Adult	147 (25.0)	49.0		153 (27.6)	51.0		300 (26.3)
Older adult	58 (9.9)	42.0		80 (14.4)	58.0		138 (12.1)
**Total**	**208 515 (100.0)**	**72.6**		**78759 (100.0)**	**27.4**		**287 274 (100.0)**

Notes: We used data from International Committee of the Red Cross (ICRC)-owned and official ICRC-partner centres only. Young child: < 5 years, child: 5–17 years, young adult: 18–34 years, adult: 35–59 years, older adult: ≥ 60 years.

An important age cohort are people of working age (18 to 59 years); overall 51.5% (147 974‬/287 274) of the service users were in this age group and this was the case for most countries. However, in Gaza Strip, Togo and Democratic Republic of the Congo, the largest proportion of service users were younger than 5 years, 50.3% (4546/9029), 37.0% (423/1142) and 29.0% (750/2587), respectively. In Algeria, older adults constituted the greatest proportion of the service users (34.8%; 72/207).

The sex distribution varied considerably across age groups in all countries and territories. However, in the young adult and adult age groups, a consistently greater proportion of men attended the rehabilitation centres compared with women in all countries and territories, except Togo where the proportions were similar.

For children receiving rehabilitation, we found a smaller, but still important, difference in sex distribution in most countries and territories with more boys than girls attending the centres; only the Gaza Strip had equal sex distribution. The same trend was seen for older adults in most places; substantially more men than women accessed services, particularly in Ethiopia (89.7% were males; 314/350). Exceptions were the Democratic Republic of the Congo where 46.0% (105/228) in this age group were males and Togo where 42.0% were males (58/138).

**Fig. 1:**
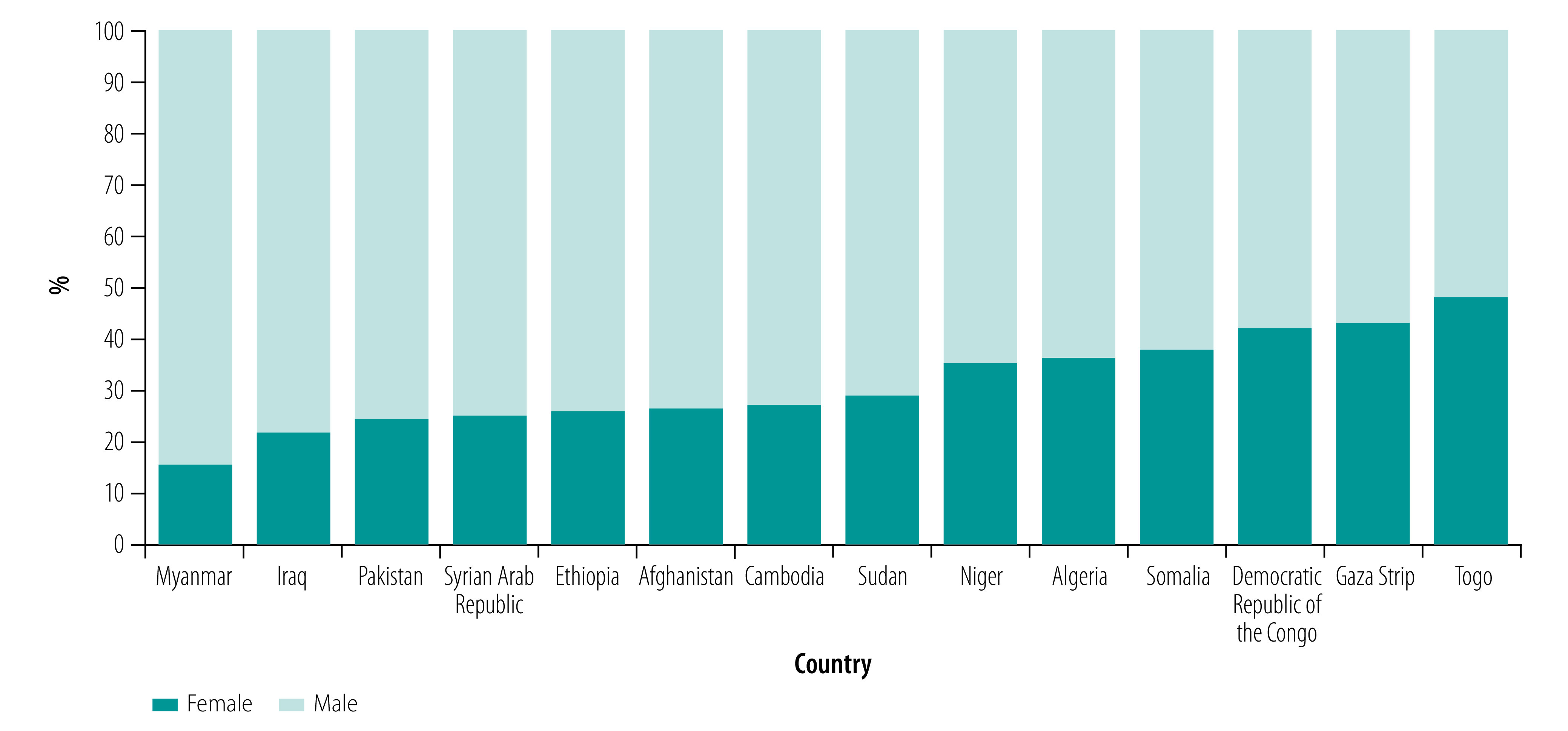
Sex distribution of people using rehabilitation services in 14 countries and territories affected by conflict, 1988–2018

Fig. 1 shows the overall sex distribution of people using rehabilitation services by country and territory, from lowest to highest proportion of females.

### Clinical data

Table 4 shows the main reasons for attending the centres over the study period. Amputation was the most common reason (33.3%; 95 574/287 274), followed by cerebral palsy (13.7%; 39 446/287 274). Less than 10% of users attended the rehabilitation centres for each of the conditions: clubfoot, fractures, hemiplegia, para- and tetraplegia and sequelae of polio. About a quarter of the service users (70 838/287 274) attended for other reasons (data available in the data repository).^14^

**Table 4:** Main condition for which service users were attending rehabilitation centres in 14 countries and territories affected by conflict, 1988–2018

Condition	Service users, no. (%)
Amputation	95 574 (33.3)
Clubfoot	12 988 (4.5)
Cerebral palsy	39 446 (13.7)
Fractures	18 952 (6.6)
Hemiplegia	11 954 (4.2)
Para- and tetraplegia	17 517 (6.1)
Sequelae of polio	20 005 (7.0)
Other	70 838 (24.7)
Total	287 274 (100.0)

Notes: We used data from International Committee of the Red Cross (ICRC)-owned and official ICRC-partner centres only.

**Fig. 2:**
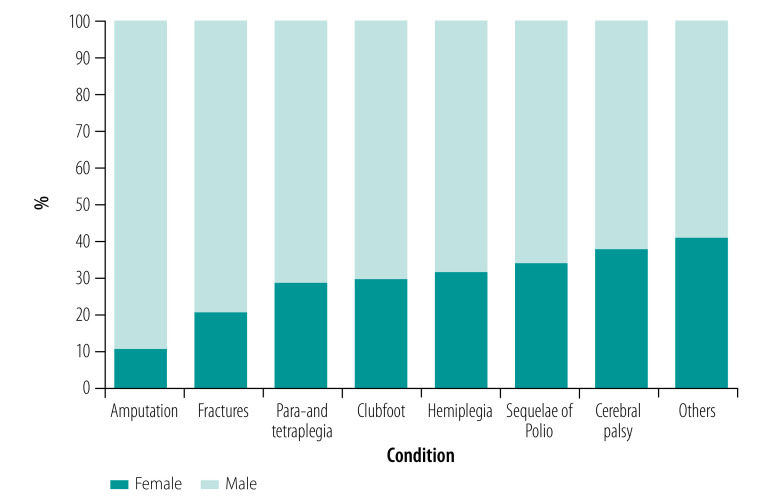
Main reasons for using rehabilitation services according to sex in 14 countries and territories affected by conflict, 1988–2018

Note: Bar representing other conditions includes 67 identified conditions.

Fig. 2 shows the sex distribution of people using rehabilitation services according to the main health condition for needing such services for all countries and territories, from the lowest to highest proportion of females.

We categorized conditions for which more than 500 users sought rehabilitation services in each country by sex and age group (data repository).^14^

Table 5 shows musculoskeletal health conditions for which service users needed rehabilitation (amputation, fractures) by sex and age. We found important differences in sex distribution especially in young adults and adults; for example, more than 90% of those presenting with an amputation in Afghanistan and Cambodia were male and more than 80% in Ethiopia, Iraq, Myanmar and Pakistan were male. Considerably more men also presented after fractures in all the countries and territories and in all age groups except for Cambodians, where 52.5% (359/684) of older adults being attended to after fractures were women.

**Table 5:** Service users attending rehabilitation centres for musculoskeletal health conditions in 14 countries and territories affected by conflict, 1988–2018

Country or territory and age group	Amputations						Fractures				
	Males			Females			Males			Females	
	No. (%)	% in age group		No. (%)	% in age group		No. (%)	% in age group		No. (%)	% in age group
**Afghanistan**	42 498 (100.0)	90.7		4 348 (100.0)	9.3		10 466 (100.0)	83.6		2 052 (100.0)	16.4
Young child	243 (0.6)	62.3		147 (3.4)	37.7		109 (1.0)	64.9		59 (2.9)	35.1
Child	4 976 (11.7)	80.3		1 219 (28.0)	19.7		1 646 (15.7)	77.0		491 (23.9)	23.0
Young adult	25 692 (60.5)	94.5		1 490 (34.3)	5.5		4 229 (40.4)	89.0		524 (25.5)	11.0
Adult	9 120 (21.5)	89.0		1 122 (25.8)	11.0		3 199 (30.6)	84.5		589 (28.7)	15.5
Older adult	2 467 (5.8)	87.0		370 (8.5)	13.0		1 283 (12.3)	76.7		389 (19.0)	23.3
**Cambodia**	16 454 (100.0)	92.0		1 427 (100.0)	8.0		2 230 (100.0)	68.8		1 009 (100.0)	31.2
Young child	40 (0.2)	65.6		21 (1.5)	34.4		27 (1.2)	67.5		13 (1.3)	32.5
Child	399 (2.4)	68.6		183 (12.8)	31.4		282 (12.6)	69.3		125 (12.4)	30.7
Young adult	7 459 (45.3)	93.1		552 (38.7)	6.9		783 (35.1)	79.7		199 (19.7)	20.3
Adult	7 927 (48.2)	93.7		530 (37.1)	6.3		813 (36.5)	72.2		313 (31.0)	27.8
Older adult	629 (3.8)	81.7		141 (9.9)	18.3		325 (14.6)	47.5		359 (35.6)	52.5
**Ethiopia**	1 300 (100.0)	83.2		262 (100.0)	16.8		ND	ND		ND	ND
Young child	4 (0.3)	66.7		2 (0.8)	33.3		ND	ND		ND	ND
Child	116 (8.9)	69.9		50 (19.1)	30.1		ND	ND		ND	ND
Young adult	415 (31.9)	77.0		124 (47.3)	23.0		ND	ND		ND	ND
Adult	544 (41.8)	87.9		75 (28.6)	12.1		ND	ND		ND	ND
Older adult	221 (17.0)	95.3		11 (4.2)	4.7		ND	ND		ND	ND
**Gaza Strip**	1129 (100.0)	79.0		301 (100.0)	21.0		ND	ND		ND	ND
Young child	21 (1.9)	58.3		15 (5.0)	41.7		ND	ND		ND	ND
Child	119 (10.5)	61.7		74 (24.6)	38.3		ND	ND		ND	ND
Young adult	481 (42.6)	90.6		50 (16.6)	9.4		ND	ND		ND	ND
Adult	337 (29.8)	76.8		102 (33.9)	23.2		ND	ND		ND	ND
Older adult	171 (15.1)	74.0		60 (19.9)	26.0		ND	ND		ND	ND
**Iraq**	6 723 (100.0)	87.1		996 (100.0)	12.9		ND	ND		ND	ND
Young child	31 (0.5)	64.6		17 (1.7)	35.4		ND	ND		ND	ND
Child	358 (5.3)	75.4		117 (11.7)	24.6		ND	ND		ND	ND
Young adult	2 360 (35.1)	91.0		234 (23.5)	9.0		ND	ND		ND	ND
Adult	3 004 (44.7)	89.3		359 (36.0)	10.7		ND	ND		ND	ND
Older adult	970 (14.4)	78.3		269 (27.0)	21.7		ND	ND		ND	ND
**Myanmar**	8 013 (100.0)	87.9		1107 (100.0)	12.1		ND	ND		ND	ND
Young child	24 (0.3)	53.3		21 (1.9)	46.7		ND	ND		ND	ND
Child	274 (3.4)	64.0		154 (13.9)	36.0		ND	ND		ND	ND
Young adult	2753 (34.4)	89.9		310 (28.0)	10.1		ND	ND		ND	ND
Adult	4214 (52.6)	90.1		464 (41.9)	9.9		ND	ND		ND	ND
Older adult	748 (9.3)	82.6		158 (14.3)	17.4		ND	ND		ND	ND
**Pakistan**	1884 (100.0)	87.2		277 (100.0)	12.8		352 (100.0)	68.3		163 (100.0)	31.7
Young child	13 (0.7)	65.0		7 (2.5)	35.0		3 (0.9)	75.0		1 (0.6)	25.0
Child	140 (7.4)	72.5		53 (19.1)	27.5		41 (11.6)	74.5		14 (8.6)	25.5
Young adult	768 (40.8)	89.2		93 (33.6)	10.8		114 (32.4)	74.5		39 (23.9)	25.5
Adult	745 (39.5)	88.1		101 (36.5)	11.9		142 (40.3)	67.0		70 (42.9)	33.0
Older adult	218 (11.6)	90.5		23 (8.3)	9.5		52 (14.8)	57.1		39 (23.9)	42.9
**Somalia**	832 (100.0)	71.2		337 (100.0)	28.8		530 (100.0)	65.0		286 (100.0)	35.0
Young child	3 (0.4)	60.0		2 (0.6)	40.0		14 (2.6)	58.3		10 (3.5)	41.7
Child	53 (6.6)	60.2		35 (10.4)	39.8		106 (20.0)	74.1		37 (12.9)	25.9
Young adult	248 (29.8)	72.9		92 (27.3)	27.1		170 (32.1)	68.8		77 (26.9)	31.2
Adult	351 (42.2)	72.8		131 (38.9)	27.2		141 (26.6)	59.7		95 (33.2)	40.3
Older adult	177 (21.3)	69.7		77 (22.8)	30.3		99 (18.7)	59.6		67 (23.4)	40.4
**Sudan**	4 974 (100.0)	77.1		1 478 (100.0)	22.9		ND	ND		ND	ND
Young child	42 (0.8)	67.7		20 (1.4)	32.3		ND	ND		ND	ND
Child	279 (5.6)	63.8		158 (10.7)	36.2		ND	ND		ND	ND
Young adult	1 295 (26.0)	75.5		421 (28.5)	24.5		ND	ND		ND	ND
Adult	2 175 (43.7)	79.2		572 (38.7)	20.8		ND	ND		ND	ND
Older adult	1 183 (23.8)	79.4		307 (20.8)	20.6		ND	ND		ND	ND
**Total^a^**	**84 717 (100.0)**	**88.6**		**10 857 (100.0)**	**11.4**		**14 968 (100.0)**	**79.0**		**3 984 (100.0)**	**21.0**

ND: not determined.

^a^ The total includes all people with the condition from all centres and countries and territories.

Notes: We used data from International Committee of the Red Cross (ICRC)-owned and official ICRC-partner centres only. For centres with fewer than 500 service users in total for a health condition, we did not determine the number or percentages. Young child: < 5 years, child: 5–17 years, young adult: 18–34 years, adult: 35–59 years, older adult: ≥ 60 years.

Table 6 shows the neurological health conditions for which service users needed rehabilitation (hemiplegia, paraplegia and tetraplegia, and sequelae of polio) by sex and age. Few people attended the rehabilitation centres with hemiplegia in all countries and territories; overall only 4.2% (11 954/28 7274). The largest groups of users with hemiplegia were adults and older adults. More men presented with hemiplegia in all the adult age groups in all countries. For paraplegia and tetraplegia, the data showed large differences in sex distribution in young adults and adults; more than 70% of users being attended to for these conditions were men. The largest proportion of service users for sequelae of polio were in the child and adult populations. About a third of users attending for sequelae of polio were females, except in users younger than 5 years in Cambodia, where 58.2% (57/98) were girls and in young adults in Sudan, where 53.6% (292/545) were women.

**Table 6:** Service users attending rehabilitation centres for neurological health conditions in 14 countries and territories affected by conflict, 1988–2018

Country or territory and age group	Hemiplegia						Para- and tetraplegia						Sequelae of polio				
	Males			Females			Males			Females			Males			Females	
	No. (%)	% in age group		No. (%)	% in age group		No. (%)	% in age group		No. (%)	% in age group		No. (%)	% in age group		No. (%)	% in age group
**Afghanistan**	5249 (100.0)	72.3		2014 (100.0)	27.7		10273 (100.0)	71.9		4005 (100.0)	28.1		8377 (100.0)	70.0		3584 (100.0)	30.0
Young child	101 (1.9)	66.4		51 (2.5)	33.6		722 (7.0)	56.6		553 (13.8)	43.4		647 (7.7)	66.2		331 (9.2)	33.8
Child	596 (11.4)	67.1		292 (14.5)	32.9		1545 (15.0)	63.0		907 (22.6)	37.0		4206 (50.2)	66.8		2089 (58.3)	33.2
Young adult	1066 (20.3)	77.1		317 (15.7)	22.9		4921 (47.9)	78.3		1361 (34.0)	21.7		2968 (35.4)	75.2		981 (27.4)	24.8
Adult	1619 (30.8)	67.5		779 (38.7)	32.5		2361 (23.0)	71.8		928 (23.2)	28.2		477 (5.7)	74.5		163 (4.5)	25.5
Older adult	1867 (35.6)	76.5		575 (28.6)	23.5		724 (7.0)	73.9		256 (6.4)	26.1		79 (0.9)	79.8		20 (0.6)	20.2
**Cambodia**	1532 (100.0)	57.5		1134 (100.0)	42.5		1066 (100.0)	67.6		510 (100.0)	32.4		1994 (100.0)	58.7		1402 (100.0)	41.3
Young child	10 (0.7)	58.8		7 (0.6)	41.2		13 (1.2)	68.4		6 (1.2)	31.6		41 (2.1)	41.8		57 (4.1)	58.2
Child	48 (3.1)	47.1		54 (4.8)	52.9		84 (7.9)	62.2		51 (10.0)	37.8		751 (37.7)	56.9		569 (40.6)	43.1
Young adult	147 (9.6)	64.5		81 (7.1)	35.5		403 (37.8)	74.5		138 (27.1)	25.5		903 (45.3)	60.5		590 (42.1)	39.5
Adult	542 (35.4)	60.6		352 (31.0)	39.4		411 (38.6)	71.1		167 (32.7)	28.9		261 (13.1)	64.0		147 (10.5)	36.0
Older adult	785 (51.2)	55.1		640 (56.4)	44.9		155 (14.5)	51.5		148 (29.0)	48.8		38 (1.9)	49.4		39 (2.8)	50.6
**Ethiopia**	ND	ND		ND	ND		ND	ND		ND	ND		640 (100.0)	61.3		404 (100.0)	38.7
Young child	ND	ND		ND	ND		ND	ND		ND	ND		37 (5.8)	57.8		27 (6.7)	42.2
Child	ND	ND		ND	ND		ND	ND		ND	ND		137 (21.4)	59.6		93 (23.0)	40.4
Young adult	ND	ND		ND	ND		ND	ND		ND	ND		342 (53.4)	59.1		237 (58.7)	40.9
Adult	ND	ND		ND	ND		ND	ND		ND	ND		96 (15.0)	70.1		41 (10.1)	29.9
Older adult	ND	ND		ND	ND		ND	ND		ND	ND		28 (4.4)	82.4		6 (1.5)	17.6
**Iraq**	ND	ND		ND	ND		431 (100.0)	70.3		182 (100.0)	29.7		670 (100.0)	66.5		337 (100.0)	33.5
Young child	ND	ND		ND	ND		27 (6.3)	45.0		33 (18.1)	55.0		7 (1.0)	77.9		2 (0.6)	22.2
Child	ND	ND		ND	ND		81 (18.8)	50.6		79 (43.4)	49.4		42 (6.3)	60.9		27 (8.0)	39.1
Young adult	ND	ND		ND	ND		188 (43.6)	81.4		43 (23.6)	18.6		361 (53.9)	67.2		176 (52.2)	32.8
Adult	ND	ND		ND	ND		111 (25.8)	84.7		20 (11.0)	15.3		236 (35.2)	65.0		127 (37.7)	35.0
Older adult	ND	ND		ND	ND		24 (5.6)	77.4		7 (3.8)	22.6		24 (3.6)	82.8		5 (1.5)	17.2
**Somalia**	775 (100.0)	68.0		365 (100.0)	32.0		ND	ND		ND	ND		ND	ND		ND	ND
Young child	16 (2.1)	53.3		14 (3.8)	46.7		ND	ND		ND	ND		ND	ND		ND	ND
Child	36 (4.6)	56.2		28 (7.7)	43.8		ND	ND		ND	ND		ND	ND		ND	ND
Young adult	85 (11.0)	52.5		77 (21.1)	47.5		ND	ND		ND	ND		ND	ND		ND	ND
Adult	328 (42.3)	76.8		99 (27.1)	23.2		ND	ND		ND	ND		ND	ND		ND	ND
Older adult	310 (40.0)	67.8		147 (40.3)	32.2		ND	ND		ND	ND		ND	ND		ND	ND
**Sudan**	ND	ND		ND	ND		ND	ND		ND	ND		779 (100.0)	53.9		667 (100.0)	46.1
Young child	ND	ND		ND	ND		ND	ND		ND	ND		91 (11.7)	61.1		58 (8.7)	38.9
Child	ND	ND		ND	ND		ND	ND		ND	ND		186 (23.9)	51.5		175 (26.2)	48.5
Young adult	ND	ND		ND	ND		ND	ND		ND	ND		255 (32.7)	46.6		292 (43.8)	53.4
Adult	ND	ND		ND	ND		ND	ND		ND	ND		186 (23.9)	61.6		116 (17.4)	38.4
Older adult	ND	ND		ND	ND		ND	ND		ND	ND		61 (7.8)	70.1		26 (3.9)	29.9
**Total^a^**	**8112 (100.0)**	**67.9**		**3842 (100.0)**	**32.1**		**12454 (10.00)**	**71.1**		**5063 (100.0)**	**28.9**		**1314 (100.0)**	**65.7**		**6861 (100.0)**	**34.3**

ND: not determined.

^a^ The total includes all people with the condition from all centres and countries and territories.

Notes: We used data from International Committee of the Red Cross (ICRC)-owned and official ICRC-partner centres only. For centres with fewer than 500 service users in total for a health condition, we did not determine the number or percentages. Young child: < 5 years, child: 5–17 years, young adult: 18–34 years, adult: 35–59 years, older adult: ≥ 60 years.

Table 7 shows paediatric health conditions for which service users needed rehabilitation (clubfoot and cerebral palsy) by sex and age. Over 80% of service users presenting with clubfoot and cerebral palsy (both conditions continue to adulthood) were younger than 18 years. For clubfoot overall, 69.9% (9084/12 988) of service users were males. Girls with cerebral palsy represented 38.1% (15 023/39 446) of users overall, with the highest proportion seen in female adults in Cambodia (49.2%; 30/61).

**Table 7:** Service users attending rehabilitation centres for paediatric health conditions in 14 countries and territories affected by conflict, 1988–2018

Country or territory, age group	Clubfoot						Cerebral palsy				
	Males			Females			Males			Females	
	No. (%)	% in age group		No. (%)	% in age group		No. (%)	% in age group		No. (%)	% in age group
**Afghanistan**	7 414 (100.0)	71.6		2 938 (100.0)	28.4		19 740 (100.0)	62.8		11 709 (100.0)	37.2
Young child	5 680 (76.6)	72.5		2 156 (73.4)	27.5		10 935 (55.4)	61.6		6826 (58.3)	38.4
Child	1354 (18.3)	68.2		630 (21.4)	31.8		7715 (39.1)	63.4		4458 (38.1)	36.6
Young adult	289 (3.9)	71.4		116 (3.9)	28.6		938 (4.8)	71.6		372 (3.2)	28.4
Adult	61 (0.8)	67.0		30 (1.0)	33.0		123 (0.6)	75.9		39 (0.3)	24.1
Older adult	30 (0.4)	83.3		6 (0.2)	16.7		29 (0.1)	67.4		14 (0.1)	32.6
**Cambodia**	543 (100.0)	56.4		420 (100.0)	43.6		1721 (100.0)	55.6		1372 (100.0)	44.4
Young child	330 (60.8)	58.8		231 (55.0)	41.2		645 (37.5)	54.9		530 (38.6)	45.1
Child	135 (24.9)	52.5		122 (29.0)	47.5		839 (48.8)	56.5		647 (47.2)	43.5
Young adult	63 (11.6)	53.8		54 (12.9)	46.2		194 (11.3)	55.7		154 (11.2)	44.3
Adult	11 (2.0)	50.0		11 (2.6)	50.0		31 (1.8)	50.8		30 (2.2)	49.2
Older adult	4 (0.7)	66.7		2 (0.5)	33.3		12 (0.7)	52.2		11 (0.8)	47.8
**Iraq**	ND	ND		ND	ND		1075 (100.0)	63.2		627 (100.0)	36.8
Young child	ND	ND		ND	ND		298 (27.7)	58.8		209 (33.3)	41.2
Child	ND	ND		ND	ND		621 (57.8)	64.8		337 (53.7)	35.2
Young adult	ND	ND		ND	ND		131 (12.2)	64.2		73 (11.6)	35.8
Adult	ND	ND		ND	ND		21 (2.0)	75.0		7 (1.1)	25.0
Older adult	ND	ND		ND	ND		4 (0.4)	80.0		1 (0.2)	20.0
**Somalia**	ND	ND		ND	ND		856 (100.0)	59.3		588 (100.0)	40.7
Young child	ND	ND		ND	ND		557 (65.1)	60.9		358 (60.9)	39.1
Child	ND	ND		ND	ND		263 (30.7)	56.2		205 (34.9)	43.8
Young adult	ND	ND		ND	ND		22 (2.6)	53.7		19 (3.2)	46.3
Adult	ND	ND		ND	ND		7 (0.8)	63.6		4 (0.7)	36.4
Older adult	ND	ND		ND	ND		7 (0.8)	77.8		2 (0.3)	22.2
**Total^a^**	**9 084 (100.0)**	**69.9**		**3 904 (100.0)**	**30.1**		**24 423 (100.0)**	**61.9**		**15 023 (100.0)**	**38.1**

ND: not determined.

^a^ The total includes all people with the condition from all centres and countries and territories.

Notes: We used data from International Committee of the Red Cross (ICRC)-owned and official ICRC-partner centres only. For centres with fewer than 500 service users in total for a health condition, we did not determine the number or percentages. Young child: < 5 years, child: 5–17 years, young adult: 18–34 years, adult: 35–59 years, older adult: ≥ 60 years.

## Discussion

The data included in the study come from countries with ongoing protracted crisis, countries hosting populations from neighbouring conflicts and post-conflict countries. Protracted conflicts last years or decades and have highly changeable patterns, including changing intensity of fighting, shifting battle lines and high, fluctuating numbers of casualties. Restricted access to and availability and awareness of rehabilitation services during and after conflict explain the large number of new attendees at the rehabilitation centres with conflict-linked disability in the overall population, even in currently peaceful places such as Cambodia.

A key finding of our study is the proportionally lower representation of females using rehabilitation services compared with males across all age groups. While we expected increased exposure to conflict-related conditions among men, a greater proportion of males than females also attended the centres for conditions not linked to conflict. Furthermore, even among people in the younger and older age groups attending the centres, who are usually not directly involved in violence, a greater proportion were males.

To interpret these findings, we need to consider the main reason for attending the centre for males and females. About one third of the people using the rehabilitation services had undergone a limb amputation. This figure reflects the physical rehabilitation programme’s original specialization in fitting prosthetics for survivors of land mines who had lost a limb.^15^ This specialization may explain the high proportions of males in mine-contaminated places, such as Afghanistan, Cambodia, Iraq and Myanmar. Amputation rates are higher in low- and middle-income countries because of road traffic incidents,^16^ poor diabetes control and insufficient health promotion for diabetes prevention and management.^17^ These underlying factors are made worse in conflict because of weakened health services and are coupled with increases in injuries as a result of violence. In our study, amputation was the most common reason for attending the centres among females, but only 11.4% (10 857/95 574) of the people seeking rehabilitation for amputation were female. Given that diabetes is the leading cause of amputation in low- and middle-incomes countries, we could expect women to present in higher proportions.^17^^–^^21^ Cerebral palsy, the second most common health condition, is a complex condition whose diagnosis requires a specialized doctor. It is likely that within the patient management system, the category of cerebral palsy included various types of developmental disorders in the absence of differential diagnostics. The worldwide prevalence of cerebral palsy is higher in children who are born preterm or with low birth weight with some evidence of a higher incidence in males than females.^22^ However, such children with cerebral palsy are unlikely to survive in conflict situations. We hypothesize that cases of cerebral palsy among girls may be underreported, especially if they have both physical and intellectual disabilities, and their rehabilitation needs are overlooked. These girls are a highly vulnerable group who are at risk of stigmatization, neglect and violence.^23^^,^^24^ Polio sequelae mainly affect children younger than 5 years,^25^ irrespective of sex;^26^ however, in our study, 65.7% (13 144/20 005) of the people attending for sequelae of polio were males. The epidemiology of spinal cord injury indicates higher rates in men than women with ratios between 2.5:1 and 5:1,^27^^–^^29^ which is just slightly higher than our ratio (2.4:1). Fractures in older adults may be age-related. Research on the prevalence of fractures in older people reports considerably higher rates in women,^30^ whereas in the older adult age group in our study, only Cambodia had marginally more female users (52.5%; 359/684). Hemiplegia in older age is also less likely to be linked to violence. Therefore, the low representation of females (32.1%; 3842/11 954) in our study is surprising when comparing it with the prevalence of stroke in low- and middle-income countries, which is reported to be higher in females.^31^^–^^33^ Studies of the sex distribution of congenital clubfoot suggest higher male proportions with reported ratios of about 2.4:1,^34^^,^^35^ which is similar to our findings (2.3:1).

Another key finding was the substantial proportion of people of working age (just over half were 18–59 years); in males, about a third were 18–34 years. This finding demonstrates the importance of rehabilitation for people with disabilities so that they have the capacity to work where possible. The finding also highlights the adverse economic impact of armed conflict, where many people sustain injury and have long-standing disability. These people may become dependent on their families or communities. They may even require extra care within the family so that an additional person may have to cease paid employment and hence be unable to help sustain the family financially. Furthermore, in societies where men have a status as breadwinners of the family and where (male) capacity is considered closely associated with physical integrity, being disabled at a working age can affect male identity and have socioeconomic and psychological consequences.^36^^–^^39^

Our data show relatively few older adults with disability attended the rehabilitation centres, but this number is likely to change in the future. Longer life expectancy and greater numbers of people with multiple morbidity and age-related disabilities ^40^ are a reality for physical rehabilitation centres.

The large numbers of young children and children attending the rehabilitation centres (39.9%; 114 698/287 274) warrant discussion. This group constitutes the future workforce of a country and, apart from clubfoot, the main conditions for which these young people were attending require lifelong resource-intensive services, which is a considerable challenge in conflict settings.

Given the overall sex difference, we require greater understanding of the reasons for these differences in access, so that rehabilitation can be adapted to meet the needs of women and girls in the environments of the countries studied. The role of women in society is important and far-reaching: often women care for several generations of a family at the same time. For example, in conflict settings, women often keep social, education and health care systems running, stabilize families, homes and communities, or participate in social and physical reconstruction after the conflict has ended.^41^ On the other hand, the connection between female gender and disability results in pronounced disadvantages, which prevent women from fulfilling their role in society. A disabled woman is very likely to be denied access to rehabilitation services, and be impoverished, unemployed and exposed to violence because of the intersectionality of her sex with a disability.^42^^,^^43^ Exclusion from rehabilitation services means women and girls with disabilities are less likely to overcome or be able to cope with their disability, which has a substantial social and economic impact. Allowing affected women and girls to benefit from comprehensive rehabilitation and be able to reach their potential contributes to achieving sustainable development goals 3 (good health and well-being) and 5 (gender equality), which in turn results in many societal and economic benefits beyond restoring function and mobility.

Our study has some strengths and limitations. Providing rehabilitation services in low- and middle-income countries, particularly during protracted conflict, is difficult.^7^ Such highly challenging settings rarely allow for the systematic collection of data on the use of rehabilitation services, which makes our multicountry study unique. The 30 years of data recording were marked by different patterns of political instability, made worse by occasional natural disasters and epidemics. Although this data set provides insight into rehabilitation needs and users in fragile settings, the data are not representative of rehabilitation needs nor of the user population of the ICRC centres. Only 38 out of 150 physical rehabilitation centres supported or run by the ICRC use the patient management system, and we could not include seven of the 38 centres as we were unable to access the data. 

ICRC support is as varied as the contexts in which the organization works, which the data collection reflects: both the data collection process and completeness varied between the physical rehabilitation centres. The number of users from whom data were collected ranged from 176 949 users in Afghanistan, where an ICRC programme has been operating for more than 30 years, to 207 users in an Algerian partner project, where the patient management system was installed just weeks before the beginning of our analysis. In some countries (Afghanistan, Myanmar and Somalia), the ICRC physical rehabilitation centres represent most functioning rehabilitation facilities. Their data may therefore reflect the national population of users of rehabilitation services and allow greater generalizability of the findings. In other countries (Cambodia, Iraq, Pakistan and Sudan), the data are less representative of population rehabilitation needs because other national and international organizations are also providing services. The ICRC closely collaborates with these organizations for referrals and to avoid duplication of services. Globally, the ICRC is the leading actor in conflict and post-conflict settings and resource-poor in terms of duration, size and scope of its operations. ICRC’s operations offer long-term support using a common approach that consists of four strategic objectives (access, quality, sustainability and participation), which are adapted to the needs and circumstances in a specific context.^8^

The lack of reference data for population prevalence in low- and middle-income countries and conflict-affected countries for the health conditions examined in our study may confound interpretation of our findings relating to the underrepresentation of women. Our results relating sex distribution of service users are discussed relative to findings from studies in similar contexts.

The database was developed from observations in the field rather than through use of an existing classification system. This approach makes the patient management system less methodical than health information systems based on the *International statistical classification of diseases and related health problems* or the *International classification of functioning, disability and health* (ICF).^44^^,^^45^ Future data collection should be based on the ICF, which also allows self-reported or observed functional limitations as the main reason for seeking rehabilitation in the absence of medical diagnostics. The main conditions for seeking rehabilitation analysed in our paper need be understood within these limitations.

Our study provides important insights on a highly vulnerable and under-researched group: people seeking rehabilitation services in fragile settings. In the absence of prevalence data, these initial findings may help guide future investigations estimating the burden of disability in conflict settings. Given the low representation of female users, our future research will build on these findings and explore gender aspects relating to access and provision of rehabilitation in conflict contexts. Research is also needed on rehabilitation outcomes in terms of functionality, reintegration and participation.
